# The Recent Progress China Has Made in Green Mine Construction, Part II: Typical Examples of Green Mines

**DOI:** 10.3390/ijerph19138166

**Published:** 2022-07-03

**Authors:** Haoxuan Yu, Shuai Li, Lifeng Yu, Xinmin Wang

**Affiliations:** School of Resources and Safety Engineering, Central South University, Changsha 410083, China; yuhaoxuan@csu.edu.cn (H.Y.); 19507486989@163.com (L.Y.); 8210183016@csu.edu.cn (X.W.)

**Keywords:** green mining, sustainability, environment, intelligent technology

## Abstract

This paper (Part II), right after the Part I, also as an information article, introduces the recent progress of “green mine construction” in China. China is a big country in resource exploitation, but there are serious problems such as hidden danger, environmental pollution and resource waste in the exploitation of mineral resources in China. Therefore, the promotion of “green” mining technology, the implementation of “green mine construction” and the promotion of small- and medium-sized mines to green non-waste mining mode transformation and upgrading are crucial measures on the road of China’s mining development, with very important practical significance. Therefore, this information paper of our Chinese mining research mainly reviews the key progress in the construction and development of green mines and introduces four typical green mine examples in China: (1) the mine with the best green environment in China: Jinhui Mining Co., Ltd., Jiuquan, Gansu Province; (2) the most advanced mine in China: Jinchuan Group Company, Jinchang, Gansu Province; (3) the oldest green mine in China: the Suichang gold mine, Lishui, Zhejiang Province; and (4) the most mechanized mine in China: the Pingshuo Coal Co., Ltd., Shuozhou, Shanxi Province. In the abstract, we claim that Part II serves as a guide to begin a conversation and to encourage experts and scholars to engage in the research of this field.

## 1. Introduction

Since 1949, China’s economy has developed rapidly, but the process of economic development has been accompanied by tremendous damage to the environment, especially in the fields of resources, agriculture and industry [1,2,3]. In order to cope with this issue, China has greatly improved its environmental situation through reform and opening up, raising people’s environmental awareness and formulating a series of environmental protection regulations [4,5,6].

For industrial production and resource exploitation activities, “green” is undoubtedly one of the key words in recent years. Many researchers have explored “green technology” in different directions, especially “green mine construction” [7,8,9,10,11] because the exploitation of resources always inevitably brings many environmental pollution problems.

Around 2018, China claimed that any resource exploitation activity must be based on the protection of human health and ecological environment, which coincides with the concept of “green mine construction” in China’s mining industry. In China, “green mine construction” is defined as a mining mode that carries out scientific and orderly mining in the whole process of mineral resources development, controls the ecological environment disturbance in the mining area and surrounding areas within a controllable range, and realizes:

(1) Ecological environment protection;
(2) Scientific mining methods;
(3) Efficient utilization of resources;
(4) Digital management.

From the above, we can conclude that in China, the four main points of green mine construction are: (1) mine environmental protection and greening; (2) optimization of mining methods; (3) introduction of advanced mining equipment; (4) commitment to the construction of intelligent mine systems.

In recent years, China has taken more efficient measures in mining environmental management and put forward the resource strategy of “green mine construction”, which has not only effectively improved China’s environmental status quo but also standardized the mining processes of China’s mines [4].

Part I [4], the last paper introduced the development process of “green mine construction” in China and the necessity of its development, narrated the current difficulties in mining in China. Therefore, also as an information article, this paper (Part II) [4] follows the introduction of the groundwater pollution of mines and key technologies in China’s “green mine construction” resource strategy mentioned in Part I, and more focuses on introducing four types of green mines in China:

1. The mine with the best green environment in China: Jinhui Mining Co., Ltd., Gansu Province (Ecological environment protection);
2. The most advanced mine in China: Jinchuan Group, Gansu Province (Digital management);
3. The oldest green mine in China: the Suichang gold mine, Zhejiang Province (Scientific mining methods);
4. The most mechanized mine in China: the Pingshuo Coal Co., Ltd., Shanxi Province (Efficient utilization of resources).

## 2. Typical Examples

### 2.1. Jinhui Mining Co., Ltd., Gansu Province

Jinhui Mining Co., Ltd. is a typical mine that attaches great importance to greening and ecological environment protection.

**Figure 1:**
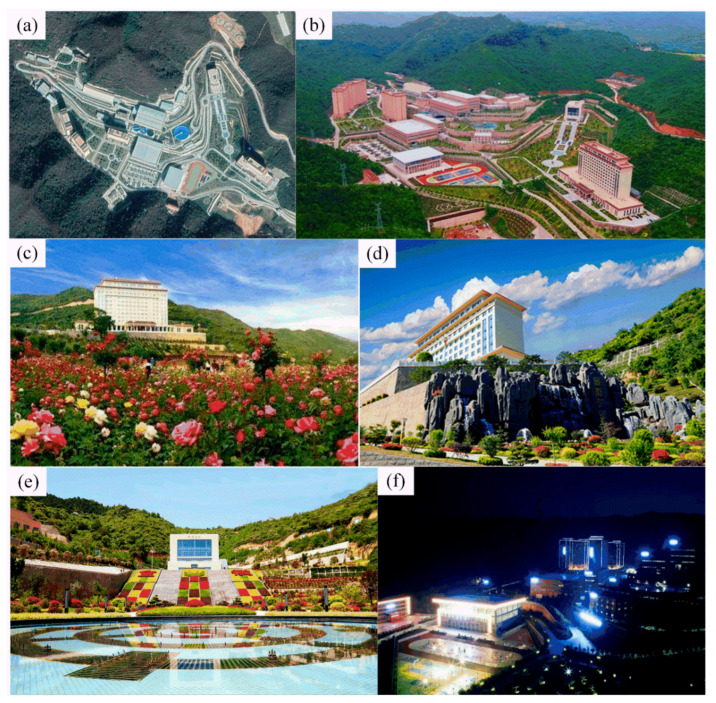
“Green mine construction” of Jinhui Mining Co., Ltd.: (**a**) satellite map of mining area (source from: www.earthol.com, access on 15 May 2022); (**b**) aerial view of the mine (source from: http://guba.eastmoney.com/news,601969,936425638.html, access on 15 May 2022); (**c**) flower gardens (source from: https://weibo.com/7136032416/HB7peCSMe?type=repost, access on 15 May 2022); (**d**) falls (source from: https://www.sohu.com/a/220186122_753295, access on 15 May 2022); (**e**) garden (source from: http://www.cnmn.net.cn/NewsShow_C.aspx?id=397069, access on 15 May 2022); (**f**) night view (source from: https://weibo.com/7136032416/HB7peCSMe?type=repost, access on 15 May 2022).

Jinhui Mining Co., Ltd., located in Northwest China’s Gansu Province, is often referred to as “The most beautiful mine in China”, with the best nature environment in China (see Figure 1a).

People used to think of this mines as a barren, bald mountain with fly ash and environmental damage. When Jinhui Mining Co., Ltd.emerged at the scene, they broke people’s cognitive (see Figure 1f): Jinhui’s mining area is surrounded by mountains and flowers (see Figure 1b).

The management staff of Jinhui Mining Co., Ltd. emphasized the ecological construction of the mining area and is committed to establishing the mining area into a life square exclusively for employees according to the construction standard of the scenic spot (see Figure 1e). In the mining area, the staff worked together to plant quite a few plants, such as brightly colored flowers (see Figure 1c), and set up many artificial landscapes, such as rockery and water (see Figure 1d).

**Figure 2:**
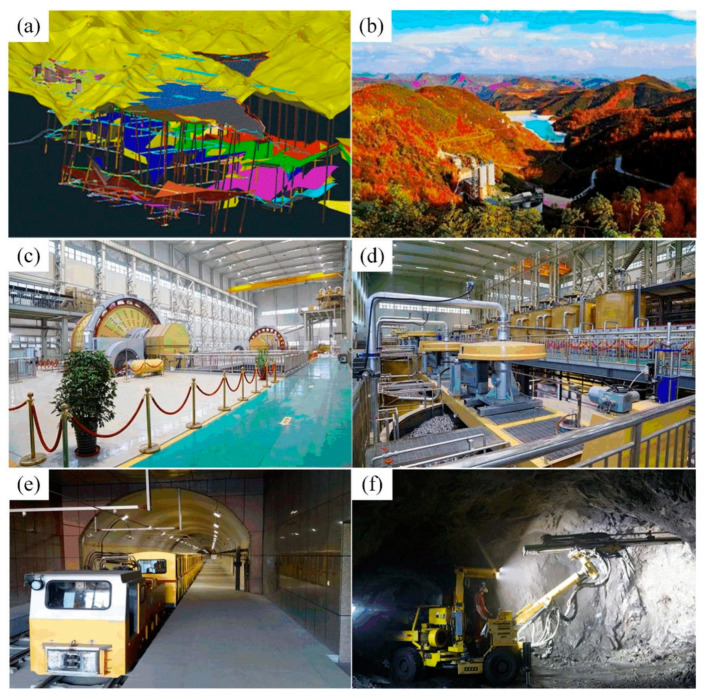
Mechanized mining situation of Jinhui Mining Co., Ltd.: (**a**) 3D digital platform of mine (source from: Jinhui Mining Co., Ltd.); (**b**) Tailings pond (source from: https://m.sohu.com/a/433359737_99917933, access on 15 May 2022); (**c**) CH420 cone crusher (source from: https://www.cnmn.com.cn/ShowNews1.aspx?id=432343, access on 15 May 2022); (**d**) KYF II-70 flotation machine (source from: http://www.gshxzf.gov.cn/show/id/184.html, access on 15 May 2022); (**e**) Underground transport car (source from: Jinhui Mining Co., Ltd.); (**f**) Downhole drilling trolley (source from: https://new.qq.com/rain/a/20210526a0dqfb00, access on 15 May 2022).

Additionally, to making great progress in environmental construction of the mining area, Jinhui Mining Co., Ltd. actively develops mining, beneficiation and other technologies, and takes the initiative to introduce high-tech mining technology:

1. In terms of environment protection, Jinhui Mining Co., Ltd. attempted to introduce a 3D deposit modeling system [12] to conduct underground exploration in combination with metallogenic mechanisms under known mine geological conditions, which improved exploration efficiency and achieved remarkable results (see Figure 2a).
2. In terms of tailings management, Jinhui Mining Co., Ltd. actively adopts the approaches of dehydration, drainage and dry pile to establish tailings, and adopts the “Three-dimensional Tailings pond drainage system” (see Figure 2b).
3. In terms of beneficiation, the innovative process of underground coarse grinding and crushing is adopted, and high-tech and sophisticated large-scale equipment is equipped, such as the CH420 cone crusher (see Figure 2c) and KYF II-70 flotation machine (see Figure 2d), which improves the beneficiation efficiency;
4. In mining, Jinhui Mining Co., Ltd. adopts a backfill mining method (subsequent backfill) to backfill the goaf with wastes (such as waste rock, tailings, metal slag) generated in the course of mining and beneficiation to improve ore recovery rate and diminish loss and dilution rate.
5. In terms of ore transportation, Jinhui Mining Co., Ltd. is committed to using a signal control system to dispatch electric locomotives (vehicles), which improves the transportation efficiency and vehicle utilization rate (see Figure 2e).

In other aspects, Jinhui Mining Co., Ltd. uses advanced trackless equipment such as a drilling trolley in the process of mining and tunneling (see Figure 2f).

### 2.2. Jinchuan Group, Gansu Province

Jinchuan Group, as a large mine in China, is famous for its digital management and production system while focusing on mining land reclamation.

**Figure 3:**
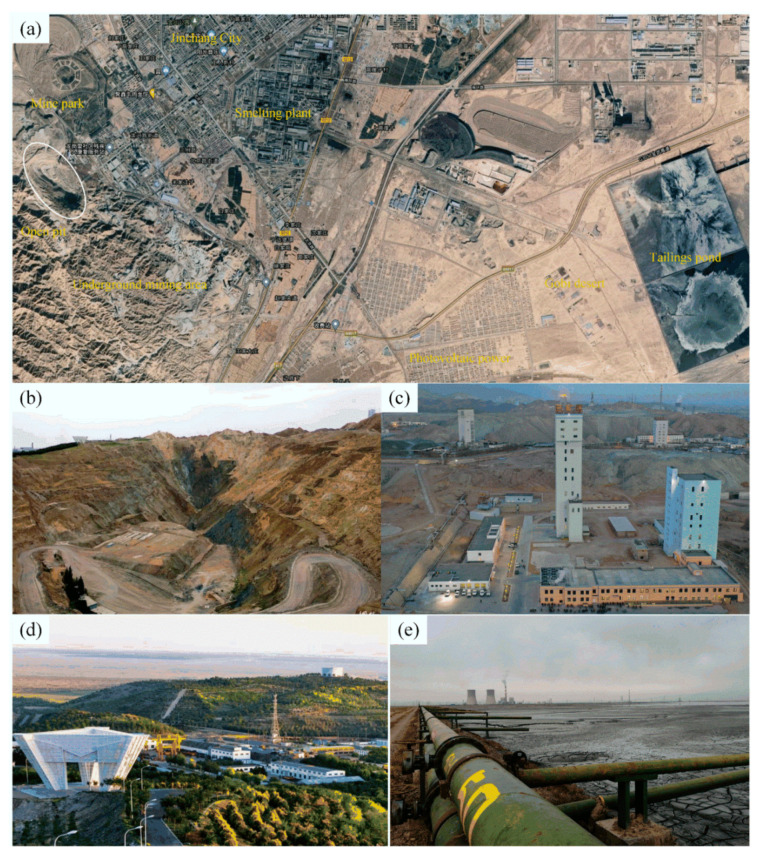
Main production facilities of Jinchuan Group: (**a**) satellite map of mining area (source from: www.earthol.com, access on 15 May 2022); (**b**) closed open-pit stopes (source from: https://www.qqai.net/mpzoqh/, access on 15 May 2022); (**c**) surface industrial sites (source from: https://www.sohu.com/na/450587881_267106, access on 15 May 2022); (**d**) national mining parks (source from: https://www.sohu.com/a/156763883_563120, access on 15 May 2022); (**e**) tailings ponds (elaborated by authors).

Jinchuan Group of Gansu Province is located in the northwest of China and is referred to as “China’s most advanced mines”, It is, within the scope of mining and technology of China, one of the most advanced, high degree of mechanization mining (see Figure 3a) [13].

At the beginning of its establishment, Jinchuan Group mainly engaged in open-pit mining (see Figure 3b). Nevertheless, with the passage of time, Jinchuan Group has gradually shifted to underground mining and achieved good benefits (see Figure 3c), with an average annual ore production of more than 4 million tons. Although the mining of nickel ore in the open air caused a certain amount of environmental damage, such as the left artificial pit, as well as the discharge of waste rock and waste soil, Jinchuan Group in a timely manner, implemented environmental protection measures, which provided incredible use of artificial mines and mining waste rock construction (Jinchuan National Mine Park), and positive results for mining area greening (see Figure 3d) [14].

**Figure 4:**
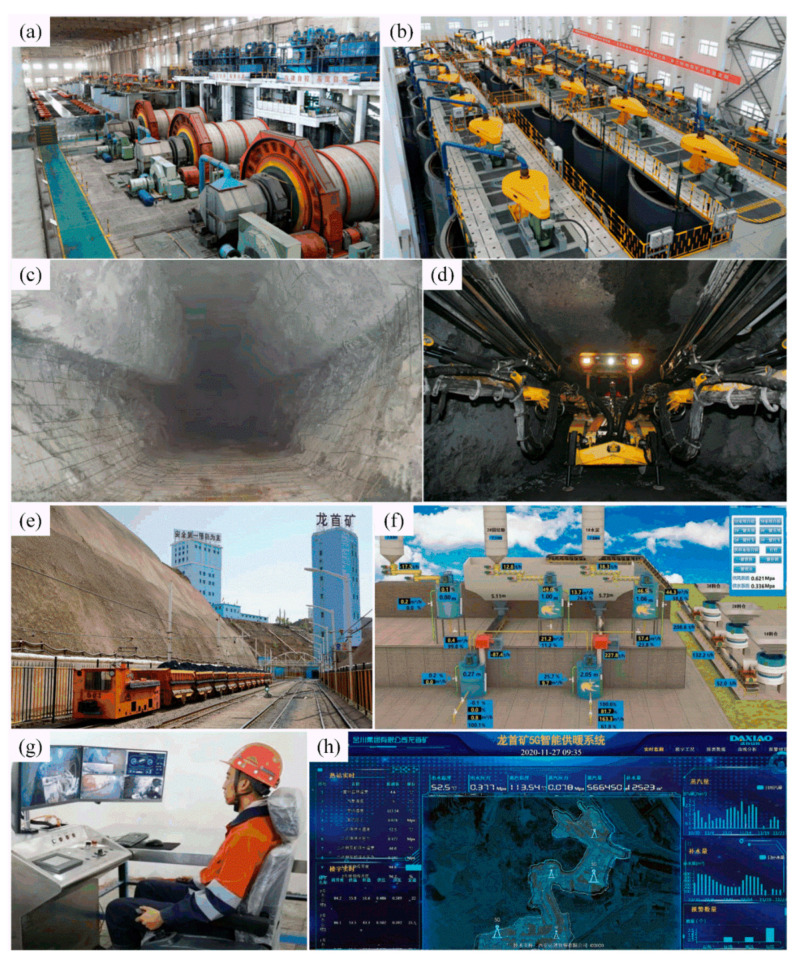
Mechanized and digital management and production systems of Jinchuan Group: (**a**) ore crushing and ball mill workshop (source from: http://m.stdaily.com/index/kejixinwen/2021-06/19/content_1158139.shtml, access on 15 May 2022); (**b**) concentrate flotation workshop (source from: https://baijiahao.baidu.com/s?id=1644992078367966371&wfr=spider&for=pc, access on 15 May 2022); (**c**) downward hexagonal approach (source from: http://www.qzsyg.com/zixun/1/34428248.html, access on 15 May 2022); (**d**) JCZY-252 wheeled full hydraulic rock drilling rig (source from: https://www.163.com/dy/article/G8015NJ60534697A.html, access on 15 May 2022); (**e**) 5G+ electric locomotive driverless (source from: https://www.sohu.com/a/440557404_120206435, access on 15 May 2022); (**f**) control interface of one-key backfill system in mine (source from: https://www.meipian.cn/3bnpg4hj, access on 15 May 2022); (**g**) JCCY-6 remote control LHD produced by Jinchuan Group (source from: http://gansu.gansudaily.com.cn/system/2021/04/25/030323276.shtml, access on 15 May 2022); (**h**) 5G intelligent heating system in mining area (source from: Jinchuan Group).

Jinchuan Group has not only made great progress in environmental construction of the mining area, but also actively developed mining and mineral processing technology and introduced advanced mining technology [15,16]:

1. In terms of mining equipment, due to the complex geological conditions of the mine and the difficulty of mining, Jinchuan Group has invested a lot of capital to introduce high-tech trackless equipment, such as a drilling trolley and scraper (see Figure 4d);
2. In terms of mining methods, Jinchuan Group adopts the backfill mining method for ore mining. According to its unique ore body and mine geological conditions, Jinchuan Group adopts the innovative hexagonal approach backfill method (see Figure 4c) to improve mining stability and solve the problem of mining waste.
3. Regarding intelligent technologies, Jinchuan Group is committed to the construction of an intelligent mine, including successively realizing an intelligent inspection robot of 5G, 5G+ rail transport unmanned (see Figure 4e), 5G+ ore truck remote control (see Figure 4g), breaking the concentrator system (see Figure 4a,b) and intelligent technologies, which solved the exploitation of intelligent degree and system running stability problems.

### 2.3. Suichang Gold Mine, Zhejiang Province

**Figure 5:**
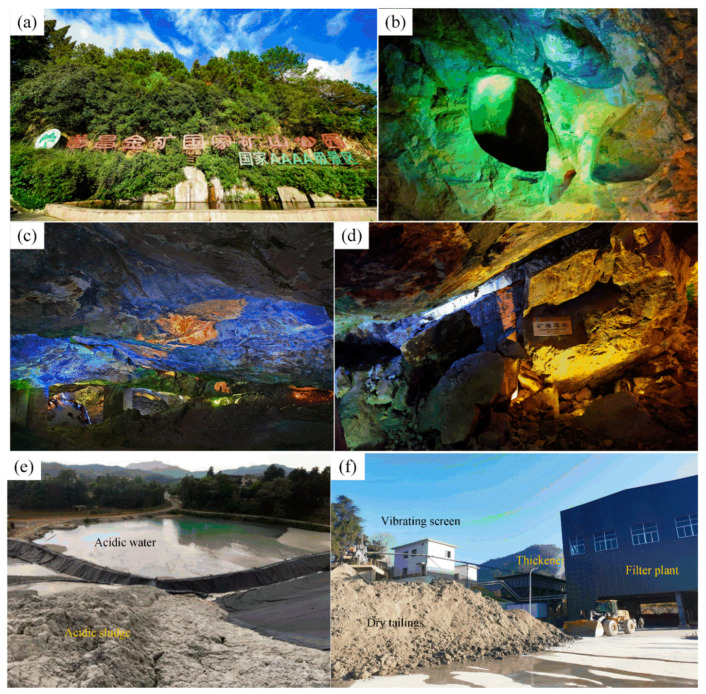
Construction of green mine at Suichang gold mine: (**a**) exterior view of the Suichang National Mine Park (source from: http://www.zzgly.cn/thread-10184-1-1.html, access on 15 May 2022); (**b**) the gold grottoes of the Tang Dynasty (source from: http://www.yidianzixun.com/article/0RrZEpJv, access on 15 May 2022); (**c**) the gold grottoes of the Song Dynasty (source from: https://www.sohu.com/a/255712356_684617, access on 15 May 2022); (**d**) mining disaster sites of the Ming Dynasty (source from: https://www.19lou.com/forum-32-thread-4401485252927652-1-1.html, access on 15 May 2022); (**e**) treatment of acid water in old goaf (elaborated by authors); (**f**) comprehensive utilization of total dehydration of tailings (elaborated by authors).

The Suichang gold mine is the oldest “green mine” in China, using a non-blasting mining method to improve production efficiency while protecting the environment. “Burn and explosion” mining is a method of mining ore in ancient China (Qin dynasty, Tang Dynasty and Ming Dynasty). It means that the rock (ore) is heated and then cooled by cold water quickly, resulting in thermal expansion and cold contraction of the ore and spalling (see Figure 5b) [17].

The Suichang gold mine, located in southwest China’s Zhejiang Province, has many traces of ancient gold mining and is considered to be the oldest gold mine in China (see Figure 5a).

The Suichang gold mine has a mining history of many hundred years, and its mining methods and techniques reached a peak in the Song Dynasty. In mineral processing, the Suichang gold mine introduced hydrodynamic crushing and screening ore. In terms of smelting, the Suichang gold mine erected the Yongfeng Silver Field, specifically for metallurgy (see Figure 5c). In the Ming Dynasty, an increasing number of manpower and resources were gradually invested into the Suichang gold mine. However, with the continuous increase of mined-out areas, the safety of mining operations was also affected. Therefore, after a mining accident (see Figure 5d), the local government of the Ming Dynasty ordered the Suichang gold mine to stop gold mining activities [18].

Although 800 years ago, there was no definition of a “green mine” in China, looking back from now, we will find that the mining of the Suichang gold mine meets “scientific mining methods” in the key elements of China’s current “green mine”.

In modern times, the Suichang gold mine has also made a lot of progress in ecological environment protection:

1. In terms of wastewater treatment, the Suichang gold mine is committed to the treatment of wastewater (acid wastewater) discharge and pollution, and actively builds sewage and wastewater treatment plants, which has reached remarkable results (see Figure 5e).
2. In terms of the environmental improvement, the Suichang gold mine is committed to the mining reclamation and greening, even planting fruit trees, corn, vegetables and other food and crops on the abandoned slope of the mining area, which improves the local economic benefits;
3. In tailings treatment, the Suichang gold mine adopts the backfill mining method to carry out mining operations, and backfills all waste rock tailings in the mining process to the underground goaf to prevent the pollution caused by tailings discharge to the surface environment. At the same time, some tailings containing a large number of heavy metal ions are stacked in a special tailings pond to avoid the pollution of groundwater caused by the backfill process.
4. In terms of tailings utilization, the Suichang gold mine has introduced an advanced tailings classification system and combined it with a tailings dehydration system (see Figure 5f) to classify (sort) tailings according to their particle sizes. Coarse (rough) particle sizes are used for construction and manufacturing, and fine particle sizes are used for backfill aggregate.

**Figure 6:**
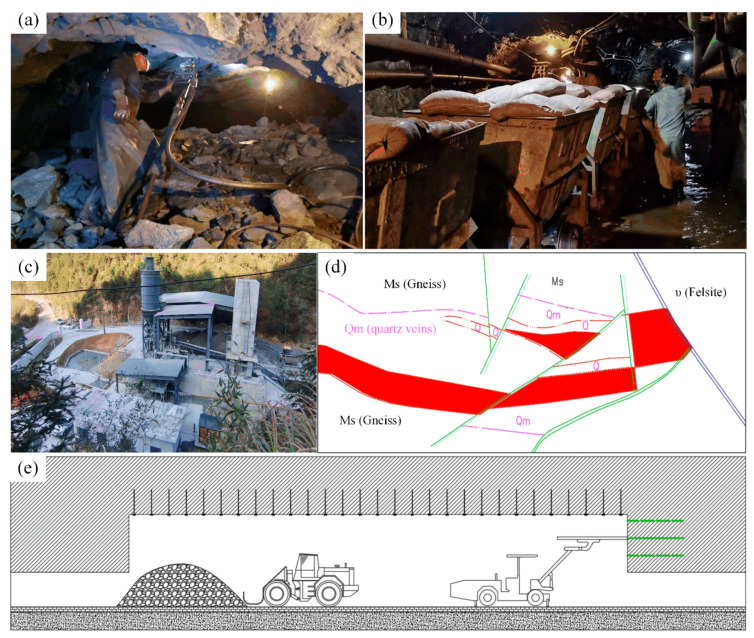
Mechanized mining in the Suichang gold mine: (**a**) inefficient pneumatic drilling; (**b**) underground transport vehicles; (**c**) tailings backfill system; (**d**) deep resources exploration; (**e**) mechanized backfill mining. (All are elaborated by authors.)

The Suichang gold mine has not only made great progress in environmental construction of the mining area, but also actively developed mechanized mining technology and introduced high-tech mining technology [19]:

1. In terms of mining technology optimization, the geological situation of the Suichang gold mine is complicated (see Figure 6d), and the rail mining mode of an air-leg drill (see Figure 6a) and mining transportation system (see Figure 6b) has been used. Now, the Suichang gold mine actively introduces trackless equipment such as the drilling platform, scraper and truck, and chooses the upward horizontal slatting and the backfill method (see Figure 6e) as the mining method, which improves the ore recovery rate.
2. In terms of backfill equipment and system optimization, the Suichang gold mine tries to establish a tailings backfill system, which backfills stopes and goaves with fine particle size tailings, improving the waste utilization rate and mining and production safety.

### 2.4. Pingshuo Coal Co., Ltd., Shanxi Province

The Pingshuo Coal Co., Ltd. is known as “the most mechanized mine in China” due to its active cooperation with the United States at the end of the 20th century and the introduction of many large and advanced mining and production equipment. However, at the same time, the Pingshuo Coal Co., Ltd. has also done very well in the land reclamation of mining areas and is the pioneer in combining mining industry and animal husbandry.

**Figure 7:**
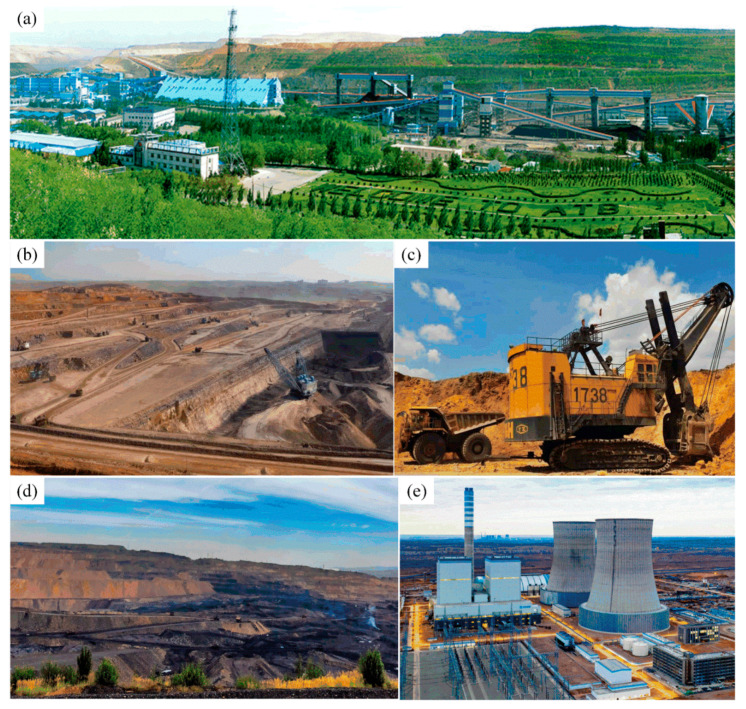
Open-pit mining in the Pingshuo Coal Co., Ltd. area: (**a**) industrial square in mining area (source from: Pingshuo Coal Co., Ltd.); (**b**) the Antaibao mining area (source from: https://www.sohu.com/a/249352885_160197, access on 15 May 2022); (**c**) heavy trucks and power shovels (source from: http://www.360doc.com/content/17/1231/17/51272721_717933400.shtml, access on 15 May 2022); (**d**) the Anjialing mining area (source from: https://www.huitu.com/photo/show/20131208/215510473200.html, access on 15 May 2022); (**e**) the eastern opencast (source from: https://new.qq.com/rain/a/20211117a06opy00, access on 15 May 2022).

The Pingshuo Coal Co., Ltd., located in Shanxi Province in northern China, is one of China’s most essential coal mining sites (see Figure 7a). The Pingshuo’s mining area is equipped with top-notch mining and production technology equipment, such as heavy trucks and power shovels (see Figure 7c). At the same time, on the basis of the advanced technical equipment, the Pingshuo Coal Co., Ltd. actively implements the modernization of information mining enterprise management; therefore, the Pingshuo Coal Co., Ltd. has become the largest open-pit mine in China, also known as one of the mines with the most sense of science and technology in China [20].

**Figure 8:**
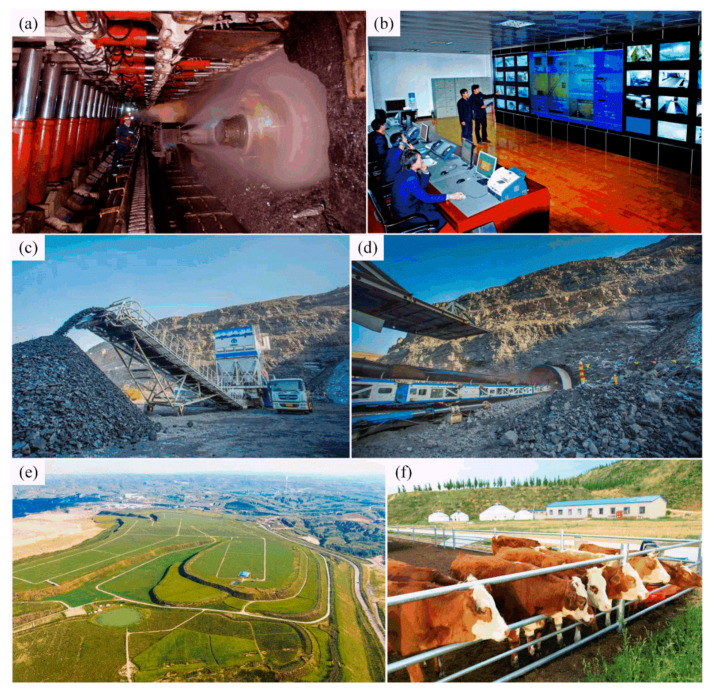
Mining conditions of the Pingshuo Coal Co., Ltd.: (**a**) fully mechanized mining face (source from: http://xjcec.com/c/2019-10-21/503848.shtml, access on 15 May 2022); (**b**) surface dispatching control room (source from: Pingshuo Coal Co., Ltd.); (**c**) edge coal mining system (source from: https://www.sohu.com/a/503659750_121123743, access on 15 May 2022); (**d**) border coal transport system (source from: https://www.sohu.com/a/503659750_121123743, access on 15 May 2022); (**e**) reclamation of the dump (source from: https://www.sohu.com/a/249352885_160197, access on 15 May 2022); (**f**) construction of modern ecological agriculture (source from: https://www.sohu.com/a/249352885_160197, access on 15 May 2022).

In the 1980s, under the gradual easing of Sino-US relations, Occidental Petroleum Company cooperated with China National Coal Development Corporation in the development of the Pingshuo Antaibao No. 1 open-pit coal mine, which soon became the largest open-pit coal mine in the world (see Figure 7b,d) [21]. In recent years, the Pingshuo Coal Co., Ltd. has paid particular attention to mining area governance and green reclamation, actively implemented “green mine construction”, actively developed animal husbandry in the mining area (see Figure 8f), and tried to plant green plants, food crops and cash crops (see Figure 7a and Figure 8e).

In addition to making great progress in environmental construction of mining area, the Pingshuo Coal Co., Ltd. also actively develops intelligent technology, so it is frequently referred to as the most mechanized mine in China [22]:

1. The east open-pit mining area of Pingshuo Coal Co., Ltd. is the most intelligent mine in Shanxi Province, even the whole of China (see Figure 7e). The Pingshuo Coal Co., Ltd., through the construction of an advanced, reasonable and intelligent 5G network control system in the east open-pit mining area, has automatic control of unmanned transport vehicles and drilling RIGS and other equipment in the east open-pit mining area;
2. At the same time, this 5G network control system is equipped with sensors, which can collect the production information of the mining area in real time through remote detection and control, and transfer the required information to the control room, thus improving the efficiency of mining operations and transportation (see Figure 8b). In addition, the Pingshuo Coal Co., Ltd. can more comprehensively coordinate the production operation of the whole mine by using the characteristics of low delay and high reliability of the 5G network, so that the mine can realize “intelligent and automatic”, which provides certain reference and reference value for the development direction of other mines in China and even the world.
3. In terms of mining method optimization, to improve the recovery rate of coal resources, the Pingshuo Coal Co., Ltd. refers to pillarless mining in metal mines and tries to adopt pillarless mining technology, which has achieved remarkable results (see Figure 8c,d). With this innovative technology, the Pingshuo Coal Co., Ltd. has successfully applied the fully mechanized top-coal caving process under complex geological conditions (see Figure 8a).

## 3. Discussion: Key technologies in “Green Mine Construction”

As mentioned above, the key elements of “green mine construction” are the following four points:

(1) Ecological environment protection:

The key technologies of “ecological environment protection” are wastewater recycling and tailings dry heap. At present, China’s water resources exploitation consumption, low recycling efficiency, heavy metal pollution and other problems are very prominent, resulting in a certain degree of water shortage, but also to the local drinking water sources and crops, and the ecological environment has a negative impact. Therefore, it is of great significance to use appropriate wastewater treatment technology to treat and comprehensively utilize mine wastewater for promoting the economic development of the mine area and its region [4]. Tailings dry heap is a kind of tailings discharge method which dehydrates tailings for dry stockpiling. It not only has lower cost, but also has less pollution and impact on the environment.

(2) Scientific mining methods:

The key technology of “scientific mining methods” is the backfill mining method. The backfill mining method is widely used in non-ferrous metal mines and precious metal mines. It can maximize the recovery of underground mineral resources, protect the surface environment and buildings, and is one of the key technologies in “green mine construction” [23].

(3) Efficient utilization of resources:

The key of “efficient utilization of resources” is to introduce advanced mining and production equipment. For example, the China Huaibei Mining Group proposed to comprehensively promote the new era of intelligent mining, introduce automatic mining technologies and equipment, and adhere to the development of “green mining” and “intelligent mining”. At present, the Huaibei Mining Group has made great achievements in intelligent coal mining, and has built an intelligent fully-mechanized coal mining working area [4].

(4) Digital management:

The key technologies of “scientific mining methods” are digital and intelligent technologies, such as 5G technology and ZigBee technology. For example, the 5G information management system and 5G+ equipment that Jinchuan Group focuses on developing are appearing more and more frequently in mining production [24]. In addition, ZigBee technology [25,26], IoT (Internet of Things) technology and other intelligent systems [27] are increasingly being used in mining operations, mine production and mine management, which are key technologies in “green mine construction”.

## 4. Conclusions

As a medium to guide readers to understand the development of China’s mining industry, this information article (Part II) introduces four typical green mines in China, namely Jinhui Mining Co., Ltd., Jinchuan Group, Pingshuo Coal Co., Ltd. and Suichang gold mine.

(1) Jinhui Mining Co., Ltd. is the mine with the best green environment in China, which corresponds to “ecological environment protection” in the key elements of “green mine construction” in China. In the introduction of Jinhui Mining Co., Ltd., this paper mainly introduces the outstanding progress made by Jinhui Mining Co., Ltd. in the greening and environmental protection of mining areas;
(2) Jinchuan Group has the most advanced mine in China, which corresponds to “digital management”, a key element of “green mine construction” in China. In the introduction of Jinchuan Group, this paper introduces the progress Jinchuan Group has made in land reclamation, and mainly introduces the digital (5G) production and management system of Jinchuan Group;
(3) The Suichang gold mine is the oldest mine in China, although 800 years ago, there was no definition of “green mine” in China. Looking back from now, we will find that the Suichang gold mine using non-blasting mining method corresponds to “scientific mining methods”, which is one of the key elements of “green mine construction” in China. This paper introduces the Suichang gold mine mining history and current development, mainly introduces the Suichang gold mine unique mining method, that is, the “burn and explosion” mining method.
(4) The Pingshuo Coal Co., Ltd.’s mine is the most mechanized mine in China. Since the beginning of last century, it has had close cooperation with the United States and introduced advanced equipment to improve the mine production efficiency, which corresponds to the key element of “efficient utilization of resources” in China’s “green mine construction”. This paper introduces the efficient mining technology of the Pingshuo Coal Co., Ltd. At the same time, this paper also introduces the progress of the Pingshuo Coal Co., Ltd. in the combination of agriculture, animal husbandry and mining industry.

We claim that this case report (Part II) serves as a guide to starting a conversation, and we hope many more experts and scholars will be interested and engage in research in this field. At the same time, we also call on relevant researchers to actively invest in the research on green mining technologies, and promote the rapid development of green mine construction, which will undoubtedly bring good news to people all over the world.

## Data Availability

Not applicable.
